# Ultrasound-Enhanced Tumor Penetration of Carrier-Free Nanodrugs for High-Efficiency Chemo-Photodynamic Therapy of Breast Cancer

**DOI:** 10.3390/jfb16060206

**Published:** 2025-06-03

**Authors:** Yun Xiang, Shiyu Liang, Ping Wang

**Affiliations:** 1 Department of Ultrasound, The Third Affiliated Hospital, Southern Medical University, Guangzhou 510630, China; 2 NMPA Key Laboratory for Research and Evaluation of Drug Metabolism, Guangdong Key Laboratory of New Drug Screening, School of Pharmaceutical Sciences, Southern Medical University, Guangzhou 510515, China

**Keywords:** photodynamic therapy, carrier-free, ultrasound, breast cancer, self-assembly

## Abstract

In recent years, chemo-photodynamic combinational therapy has become increasingly popular in treating breast cancer. However, the limited accumulation of nanodrugs into tumors (less than 1% of the injected dose) impacts therapeutic efficacy to an extreme extent. Herein, the photosensitizer Chlorin e6 (Ce6) and the chemotherapeutic drug rhein were self-assembled to form a carrier-free nanodrug (RC NPs) with good stability and a high drug loading rate (nearly 100%). In vitro, the phototoxicity of RC NPs resulted in a mere 17.8% cell viability. Ultrasound (US) irradiation was applied to increase the permeability of tumor blood vessels, thus greatly enhancing the drug accumulation of RC NPs in tumor tissues (1.5 times that of the control group). After uptake by tumor cells, Ce6 could produce a significant amount of reactive oxygen species (ROS) when exposed to laser irradiation, while rhein could inhibit tumor cell proliferation and affect mitochondrial membrane potential, inducing tumor cell apoptosis through the mitochondria-dependent apoptosis pathway, thus effectively realizing the combined effect of PDT and chemotherapy. The final tumor inhibition rate reached 93.7%. Taken together, RC NPs strengthen the enhanced permeability and retention (EPR) effect when exposed to US irradiation and exhibit better tumor suppression, which provides new insights into chemo-photodynamic combination treatment for clinical breast cancer.

## 1. Introduction

Breast cancer has the highest occurrence rate in female cancer patients, accounting for about 16.7% of new cases, and is also the primary reason for mortality [1,2]. Triple-negative breast cancer (TNBC) is a highly aggressive subtype of breast cancer, known for its elevated risk of disease progression and reduced survival rates [3]. While chemotherapy remains the cornerstone of therapy for early-stage and advanced TNBC, alongside traditional methods like surgery and radiotherapy [4,5], its effectiveness is often hampered by the adverse effects of chemotherapy medications and the development of drug resistance resulting from repeated drug administration [6]. Hence, there is a pressing necessity to explore a more promising therapeutic strategy for addressing TNBC while minimizing adverse effects.

The combination of chemotherapy with various anticancer strategies, such as immunotherapy [7], photothermal therapy [8], and photodynamic therapy [9], has drawn much attention because it breaks through the limited effectiveness of single-agent chemotherapy [10]. Among them, PDT kills tumor cells by converting molecular oxygen into highly toxic ROS under laser irradiation with a photosensitizer, which has the advantages of spatiotemporal controllability and non-invasiveness [11]. The utilization of active ingredients from traditional Chinese medicines, such as rhein, has shown promising therapeutic outcomes in cancer treatment [12,13,14]. Rhein, an anthraquinone compound primarily derived from Polygonum multiflorum Thunb and rhubarb [15], has inhibitory effects on cancer cell proliferation, including tongue cancer cells (SSC-4) [16], lung cancer cells (A-549) [17], nasopharyngeal carcinoma cells [18], and promyelocytic leukemia cells (HL-60) [19]. Recent research has demonstrated significant inhibitory effects of rhein on the growth of 4T1 breast cancer xenografts in mice, showing its potential in cancer therapy [20]. Rhein achieves this by influencing the potential of the mitochondrial membrane and inducing cell death through the mitochondrial-mediated apoptosis pathway [21,22,23]. However, rhein has a low bioavailability due to its hydrophobicity, which limits its clinical application.

Recently, nanomedicines without carriers, created by the self-assembly of chemotherapy drugs and photosensitizers, were shown to be able to accumulate preferentially at tumor sites due to the EPR effect and showed great synergistic therapeutic effects in a variety of cancers [24,25]. Compared with traditional nanomedicines, carrier-free nanomedicines exhibit a high drug-loading capacity and simple preparation process, thereby avoiding carrier-induced toxicity and immunogenicity [26]. However, the efficacy of these nanodrugs is hindered by factors such as tumor vascular heterogeneity and high interstitial pressure, resulting in less than 1% of the injected dose reaching the tumor tissue [27]. Enhancing the accumulation of nanoparticles in tumors is crucial for the efficacy of combination therapy for TNBC.

A variety of strategies have been developed to enhance drug-delivery efficiency, such as the design of tumor-microenvironment-adjustable or size-variable nanomedicine to promote the penetration of nanomedicines by disrupting the tumor’s dense matrix [28] or changing the size of nanoparticles [29]. The surface properties of nanoparticles have also been optimized to prolong blood circulation and increase tumor targeting and cross-cell transport abilities [30]. However, these strategies rely heavily on the design of nanocarriers, which are complex in synthesis and low in drug-loading rates and can exhibit significant toxicity and side effects, limiting their clinical application. As a common means of clinical diagnosis and treatment, ultrasound (US) offers advantages such as being non-invasive, simple, and capable of deep tissue penetration, making it a useful tool for promoting nanodrug delivery. It has been reported that US irradiation could increase the permeability of tumor blood vessels and promote the penetration depth and drug concentration of nanodrugs in tumor cells, thereby achieving ideal therapeutic effects [31,32,33].

**Scheme 1:**
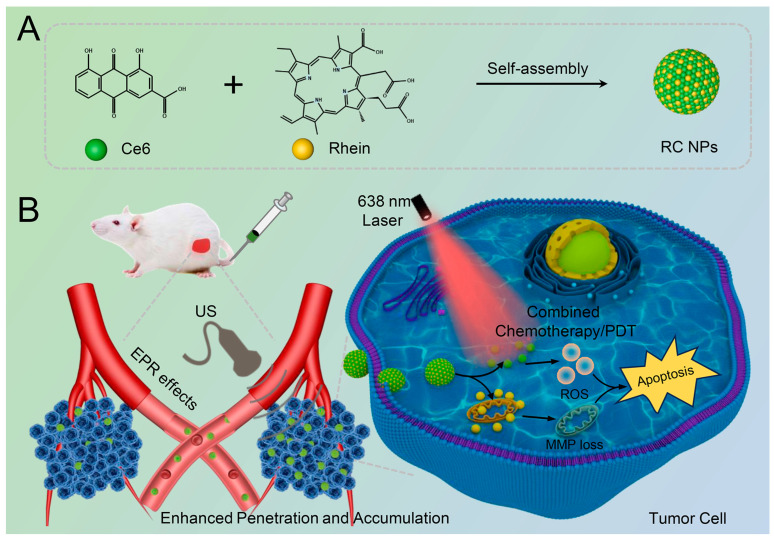
Schematic illustration of the preparation process of RC NPs and its antitumor therapeutic mechanism. (**A**) The chemical structures of rhein and Ce6, as well as the self-assembled RC NPs. (**B**) The process of using RC NPs in tumor therapy. After intravenous injection, the tumor site was irradiated with US to promote the penetration and accumulation of RC NPs in the tumor tissue, followed by laser irradiation to achieve the combined effects of chemotherapy and photodynamic therapy (Scheme 1 was created using CINEMA 4D R19 and Adobe Photoshop R2021 tools).

Herein, a carrier-free nanodrug (designated as RC NPs) for the chemo-photodynamic therapy of breast cancer was prepared by self-assembly between the chemotherapeutic agent (Rhe) and photosensitizer (Ce6) (Scheme 1A). The synthesized RC NPs exhibited a high drug-loading rate and demonstrated favorable dispersion stability. After intravenous injection, RC NPs showed significant tumor accumulation and deep penetration upon US irradiation (Scheme 1B). Upon internalization by tumor cells, Ce6 could produce a large number of ROSs under laser irradiation for PDT, while rhein could reduce mitochondrial membrane potential and induce cell apoptosis through the mitochondrial-dependent apoptosis pathway, thus effectively realizing the combined PDT and chemotherapy. Our study demonstrated that this US combined with a carrier-free nanodrug treatment strategy could effectively increase the drug concentration in the tumor tissue and achieve better therapeutic effects for chemo-photodynamic therapy in breast cancer with high biosafety.

## 2. Materials and Methods

### 2.1. Materials

Chlorin e6 (Ce6) was bought from Frontier Scientific (Logan, Utah). Rhein was bought from Macklin Biochemical Technology Co., Ltd. (Shanghai, China). 3-[4,5-dimethylthiazol-2-yl]-2,5-diphenyltetrazoliumbromide (MTT), annexin V-FITC/PI cell apoptosis kit and Calcein-AM/PI cell viability/cytotoxicity detection kit were bought from Yeasen Biotechnology Company (Shanghai, China). 2′,7′-dichlorofluorescin diacetate (DCFH-DA) and mitochondrial membrane potential detection kit (JC-1) were bought from Beyotime Biotechnology Co., Ltd., (Shanghai, China). All additional chemicals were of analytical quality and used with no other processing.

### 2.2. Preparation and Characterization of RC NPs

The nanoprecipitation approach was used to create RC NPs through the self-assembly of rhein and Ce6 at a 3:1 molar ratio. Initially, rhein and Ce6 powders were dissolved in dimethyl sulfoxide (DMSO), and then solutions of rhein (solution A) and Ce6 (solution B) with a final concentration of 20 mM were prepared, respectively. Subsequently, 45 μL of solution A and 15 μL of solution B were mixed and stirred for 4 h. The obtained solution was then dispersed in 2 mL of ultrapure water under sonication and subjected to overnight stirring. The next day, a dialysis bag (MWCO: 3500 Da) was used for dialysis for 12 h to remove the unassembled free drugs. Finally, the nanodrugs in the dialysis bag were centrifuged at 1200× *g* for 10 min to collect the supernatant. The RC NPs solution was stored at 4 °C for later usage.

Thereafter, the particle size and polydispersity index (PDI) of samples were detected using dynamic light scattering. The nanoparticles’ morphology was examined using transmission electron microscopy (TEM), while its stability was assessed by incubation in water, PBS, and 10% FBS over varying durations.

### 2.3. Exploration of Self-Assembly Mechanism

To explore the noncovalent interactions for the formation of RC NPs, RC NPs were incubated with 0.2% SDS and varying contents of NaCl solution (0, 0.5, 1.0, and 1.5 M), and then a Shinadu UV-2600 spectrophotometer was utilized in order to accurately detect the fluctuations in the UV-vis absorption spectra. Additionally, the spectral changes of RC NPs dispersed in water and DMSO were also examined.

### 2.4. Cell Culture

The HUVEC cells were cultured in DMEM medium with 1% antibiotics and 10% FBS, while mouse breast cancer cells (4T1) were cultured in RPMI-1640 medium with the same supplements. The cells were cultivated in an environment containing 5% carbon dioxide at a temperature of 37 °C.

### 2.5. Cellular Uptake

In 24-well culture plates with a cover glass at the bottom, 4T1 cells were seeded and left to culture overnight. Subsequently, they were cultured in a medium containing RC NPs and Ce6 (where Ce6 concentration was 5 μM) for 1, 2, 4, and 6 h, respectively. The cells were carefully rinsed with PBS after incubation and fixed for 20 min with 500 μL of 4% paraformaldehyde solution. Following the fixation process, the cell nuclei were stained with an anti-fluorescence quenching agent that contained DAPI. The intracellular fluorescence was then visualized using confocal microscopy (CLSM) (LSM 880, Carl Zeiss, Oberkochen, Germany). Meanwhile, the intracellular fluorescent intensity was examined based on flow cytometry. 4T1 cells were seeded in 6-well plates and left to incubate overnight. Subsequently, these cells were exposed to RC NPs and Ce6 for 1, 2, 4, and 6 h. Flow cytometry was employed to measure the intracellular fluorescence following cell collection (BD LSRFortessa X-20).

### 2.6. Intracellular ROS Generation

DCFH-DA was applied as a fluorescent probe to monitor intracellular ROS generation. Initially, after seeding 4T1 cells into 24-well plates, they were cultured for 12 h and then given different treatments, including PBS, Rhe, RC NPs, PBS+L, Ce6+L, and RC NPs+L. For each treatment, Ce6 (0.5 μM) and rhein (2 μM) concentrations were used, and the cells were left to grow for 4 h. Subsequently, cells were rinsed with PBS and cultured in basic media containing DCFH-DA (10 μM) for 30 min. The light groups were then irradiated via a 630 nm laser panel (120 mW/cm^2^) for 1 min. Finally, intracellular ROS was visualized by fluorescence inverted microscopy (FIM) (Axio Observer).

### 2.7. In Vitro Cytotoxicity

For cytotoxicity assessment, the MTT assay was employed. To investigate dark toxicity, 4T1 cells were seeded in 96-well plates and left to incubate for 12 h. Following this, the cells were incubated for another 24 h with fresh media that contained Ce6 and rhein at varying concentrations (0 to 0.5 μM and 0 to 2 μM, respectively). Then, 20 μL of MTT solution was applied to every well and incubated for 4 h in darkness. After removing the original medium, 200 μL of DMSO solution was added to each well, and the OD at 490 nm was detected via microplate reader (Thermo, Waltham, MA, USA). Similar to the dark toxicity assay, the phototoxicity assay involved irradiating the sample with a 630 nm laser panel (120 mW/cm^2^) for 1 min at 4 h after drug addition, followed by an additional 20 h of incubation.

### 2.8. Live/Dead Cell Staining Assay

4T1 cells were seeded in 24-well plates and left to incubate for 12 h. The medium was replaced by fresh medium containing Rhe, RC NPs, and Ce6 (Ce6: 0.5 μM, rhein: 2 μM). After incubation for 4 h, the light groups (PBS+L, Ce6+L, and RC NPs+L) were exposed to a 630 nm laser panel (120 mW/cm^2^) for 1 min, and then the incubation was continued for 1 h. Finally, the cells in various groups were treated with Calcein-AM (2 μM) and PI (4.5 μM) for 30 min. Intracellular fluorescence was visualized using FIM (Axio Observer).

### 2.9. Apoptosis Assay

Annexin V-FITC/PI method was employed to analyze apoptosis in 4T1 cells based on flow cytometry. The 4T1 cells were seeded in 6-well plates and left to incubate overnight, and then fresh medium containing Rhe, RC NPs, and Ce6 (rhein: 2 μM, Ce6: 0.5 μM) was added, following 4 h of incubation. Among them, the light groups (PBS+L, Ce6+L, and RC NPs+L) were irradiated via a laser panel (120 mW/cm^2^) for 1 min, and incubation continued for 1 h. Finally, the cells were stained with Annexin V-FITC and PI based on relevant kit instructions, and the fluorescence was then measured.

### 2.10. Detection of Mitochondrial Membrane Potential

When mitochondria are damaged, their membrane potential decreases; the changes were assessed using JC-1 staining, and with the decrease of mitochondrial membrane potential, mitochondrial fluorescence gradually changed from red to green fluorescence. 4T1 cells were incubated for 12 h in 24-well plates. After cells were divided into 6 groups, various drug formulations were added: (1) PBS, (2) Rhe, (3) RC NPs, (4) PBS+L, (5) Ce6+L, and (6) RC NPs+L (rhein: 2 μM, Ce6: 0.5 μM). After 4 h of incubation, cells in the light groups were exposed to laser irradiation for 1 min, followed by a 1-h incubation period before conducting JC-1 staining. Finally, the changes in mitochondrial membrane potential were identified using CLSM.

### 2.11. Ultrasonic Facilitated Penetration Detection In Vitro

To verify that the US could promote RC NPs to penetrate through tumor blood vessels, HUVEC cells were employed to simulate the tumor blood vessel barrier in vitro. Initially, HUVEC cells were seeded and incubated in a transwell upper chamber (2 × 10^4^/well), while 4T1 cells were seeded in 24-well plates. When HUVEC cells had grown and covered the bottom of the upper chamber, they were transferred to 24-well plates in which 4T1 cells were cultured. After that, a new medium with RC NPs (Ce6: 5 μM) was put into the upper chamber. It was then exposed to US irradiation (1.0 MHz, 0.3 W/cm^2^) for 1 min. The transwell insert was then removed and continued incubation for 4 h. The group that did not receive US treatment was designated as the control group. Finally, 4T1 cells in 24-well plates were harvested, and flow cytometry was applied to detect the passage of RC NPs through the vascular barrier after US irradiation.

Also, to make sure that US improved the penetration of RC NPs in vitro, 3D tumor spheres were made to show how cell–cell and cell–matrix interactions affect the penetration of RC NPs. Initially, 70 μL of 2.5% agarose solution was added to a 96-well plate when hot. After sufficient cooling, the surface formed a depression to prevent cell adhesion. Subsequently, 150 μL of medium containing 4T1 cells (500 cells/well) was added to each well, shaken evenly, and placed in an incubator. Half of the medium was replaced every other day. After 2 weeks, the 3D tumor spheres with a diameter of about 200 μm were identified, selected, and administered with fresh medium containing RC NPs (Ce6: 5 μM). The US treatment group received US irradiation at a frequency of 1.0 MHz with a duty cycle of 50% and intensity of 0.6 W/cm^2^ for 2 min. Subsequently, the drugs were incubated with the tumor spheres for 4 h. Tumor spheres were finally washed with PBS and transferred to confocal laser small dishes. Fluorescence in the tumor spheres was observed using CLSM.

### 2.12. Hemolysis Test of RC NPs

The female BALB/c mice utilized in the research were purchased from the Animal Experiment Center of Southern Medical University. The animal studies were conducted in accordance with the guidelines of the ethics committee of the Animal Experiment Centre of Southern Medical University (No. 00251499; approval date: 1 December 2020).

In order to carry out animal experiments on RC NPs, it is necessary to first verify the safety of nanodrugs through hemolysis experiments. Healthy BALB/c mice were anesthetized, and fresh blood was sampled via heart puncture. Pure erythrocytes were obtained after centrifugation and washing with PBS, and 4% (*v*/*v*) red blood cell suspension was prepared. A mixture was created by combining 250 μL of red blood cell suspension with a solution of RC NPs at varying concentrations (Ce6: 5, 10, 20, 25, and 50 μM). After centrifugation, 100 μL of supernatant was transferred to a 96-well plate. All samples were then analyzed for absorbance at 545 nm using a microplate reader. (Positive control group: red blood cell suspension mixed with ultrapure water; negative control group: red blood cell suspension mixed with PBS).

### 2.13. Fluorescence Imaging In Vivo

To create tumor-bearing mice models, subcutaneous injections of 4T1 cell suspension (2 × 10^6^ cells) were administered to the right subdermal dorsal region of BALB/c nude mice. Once the tumor volume reached around 200 mm^3^, the mice were divided into two groups and injected with 0.1 mL of Ce6 and RC NPs solution (Ce6 dose: 3 mg/kg), respectively. Isoflurane gas anesthesia was then applied, followed by performing in vivo fluorescence imaging of tumor-bearing mice at specified time intervals (0, 2, 4, 6, 12, and 24 h) using IVIS Lumina II (Seattle, WA, USA). We euthanized the mice 24 h after injection and collected their tumors for later experiments.

To confirm the ability of US to enhance the penetration and accumulation of RC NPs in vivo tumors, a BALB/c nude mouse model with bilateral 4T1 tumors was constructed. When the tumor volume had grown to approximately 200 mm^3^, the RC NP solution (Ce6 dose: 1.5 mg/kg) was injected. After 30 min, the right tumor was irradiated with US (1.0 MHz, 50 duty cycle, 0.6 W/cm^2^) for 10 min. The left tumor was not irradiated with US as a control. Six hours later, the anesthetized mice underwent in vivo fluorescence imaging to assess and compare the distribution of drug fluorescence in the tumors on both sides. Afterward, the tumors on both sides were excised for frozen sections and imaged using CLSM.

### 2.14. Antitumor Study In Vivo and Biosafety Assay

4T1 cells were injected into the right subdermal dorsal region to create a tumor-bearing animal model. Treatment began after the tumor volume was about 90 mm^3^. They were divided into 7 groups (*n* = 5), namely PBS, PBS+US+L, Rhe, RC NPs, Ce6+L, RC NPs+L, and RC NPs+US+L. Based on the reported dosage (2 mg/kg) of carrier-free nanomaterials associated with Ce6 utilized in animal studies, we administered a dose of 1.5 mg/kg to each mouse for preliminary trials, resulting in a favorable therapeutic outcome. The molar ratio of rhein to Ce6 in the produced RC NPs is around 4:1, resulting in a comparable rhein dosage of approximately 2.9 mg/kg. The mice received intravenous injections of the specified drugs (Ce6 dose: 1.5 mg/kg, rhein dose: 2.9 mg/kg). The US treatment groups were exposed to US (1.0 MHz, 50 duty cycle, 0.6 W/cm^2^) for 10 min after 30 min post-injection, while the light group was irradiated via laser with an intensity of 0.5 mW/cm^2^ for 10 min, 6 h post-injection. Each group of mice received therapy three times: on the day of treatment and on days 2 and 4 following the initial treatment. On the 14th day, we euthanized the mice and collected the major organs and tumor tissues for HE staining or TUNEL immunofluorescence detection.

Healthy female BALB/c mice were divided into three groups (*n* = 3): the PBS group, the 1-day-after-administration group, and the 7-day-after-administration group. The mice were administered with RC NPs solution (Ce6 dose: 3 mg/kg, rhein dose: 5.8 mg/kg) at predetermined time points, and blood was gathered for routine blood biochemical tests.

### 2.15. Statistical Analysis

The experiment’s quantitative data are presented as mean ± standard deviation (m ± SD), and statistical differences between the groups were analyzed using ANOVA (SPSS 23). A *p*-value of <0.05 was considered significant, with the following criteria: NS (no significant difference), *, **, and *** representing *p* < 0.05, *p* < 0.01, and *p* < 0.001, respectively.

## 3. Results and Discussion

### 3.1. Preparation and Characterization of RC NPs

**Figure 1:**
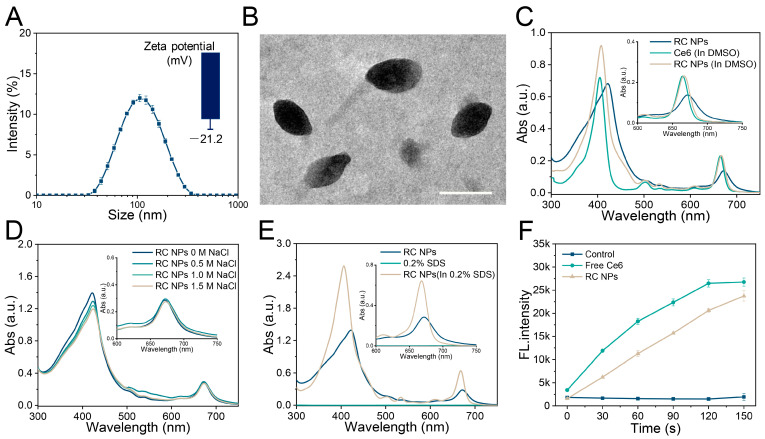
Characterizations of RC NPs. (**A**) Particle size distribution and zeta potential of RC NPs at the feeding ratio of 3:1. (**B**) TEM image of RC NPs. Scale bar: 100 nm. UV−vis absorbance spectrum of RC NPs in the presence or absence of (**C**) DMSO, (**D**) NaCl, and (**E**) SDS (0.2%, *w*/*v*). (**F**) ROS production in various solutions with laser irradiation, measured by multifunctional microplate reader using DCFH as a probe. The error bars indicate means ± SD, *n* = 3.

The self-assembled carrier-free nanoparticles not only improve the bioavailability of free hydrophobic drugs but also avoid the toxicity of additional carriers. To obtain optimal nanoparticles, RC NPs were synthesized by nanoprecipitation with Rhe and Ce6 at various molar ratios (Rhe/Ce6 = 5:1, 3:1, and 1.5:1) (Table S1). The nanoparticles formed under different feeding ratios were measured by dynamic light scattering. It was found that RC NPs with Rhe/Ce6 = 3:1 have a smaller particle size (100.6 ± 1.8 nm) and better polydispersity index (PDI). Furthermore, the zeta potential of this nanoparticle was approximately −21.2 ± 0.6 mV, which was favorable for reducing the adsorption of proteins in the blood circulation [34] (Figure 1A). TEM showed that RC NPs were spindle-shaped (Figure 1B), with nano-spindles being particularly conducive to cellular uptake and accumulation in the tumor [35]. Furthermore, the particle size distribution of RC NPs was monitored for 7 days, and no significant changes were found (Figure S1A), proving its favorable stability in water. Similarly, RC NPs also maintained stable particle size and PDI in phosphate buffer (PBS) and only showed a slight increase in particle size under 10% fetal bovine serum (FBS) (Figure S1B,C), which may be attributed to protein corona on the nanoparticle surface [36]. This stability made it possible for RC NPs to be used in biomedical applications. Since RC NPs were created through the self-assembly of two pure drug molecules, they had very high drug-loading rates. The contents of Ce6 and Rhe in RC NPs were 33.97% and 66.03%, detected by UV-vis spectrum and HPLC, respectively [25] (Figure S2).

To investigate the mechanism of self-assembly, RC NPs were subjected to various conditions, and the resulting changes in UV-vis spectra were detected. In Figure 1C–E, when comparing free Ce6 and RC NPs dissolved in DMSO, the characteristic peak of RC NPs in water was broader and redshifted about 16 nm, and the characteristic absorption peak of RC NPs did not change with the increase in NaCl concentration but changed significantly in the presence of 0.2% SDS. The results suggested that the self-assembly of Rhe and Ce6 was primarily driven by intermolecular hydrophobic and π-π stacking interactions [9]. Subsequently, the in vitro ROS generation capability of RC NPs was assessed using dichrofluorescein (DCFH) as a fluorescent probe (Figure 1F). Both Ce6 and RC NPs could generate a substantial quantity of reactive oxygen species when exposed to laser irradiation. RC NPs could be utilized for subsequent anti-tumor therapy due to their favorable photodynamic properties.

### 3.2. Cellular Uptake and In Vitro Antitumor Efficiency of RC NPs

**Figure 2:**
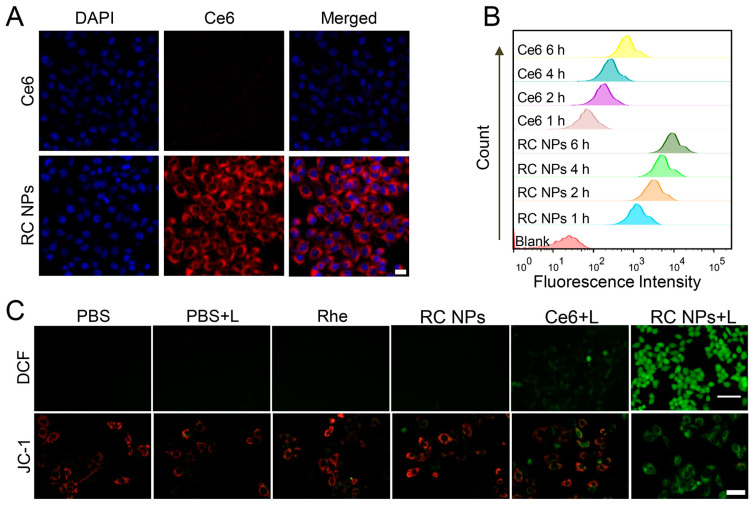

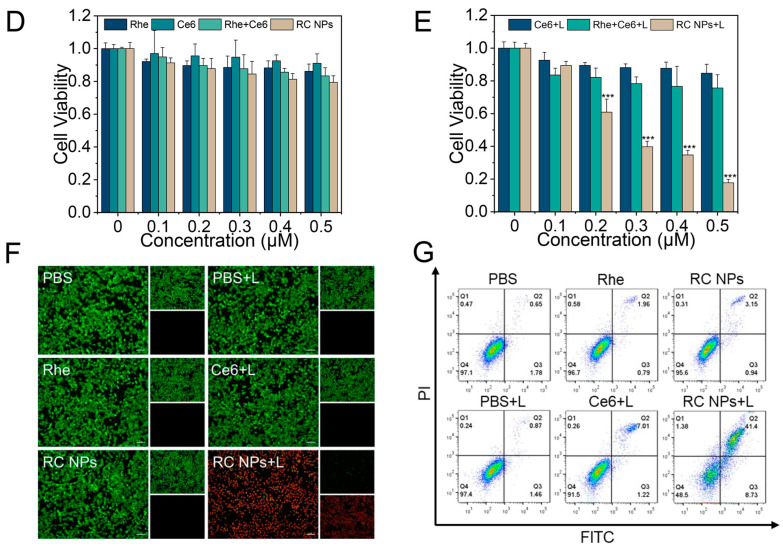
Cellular uptake and in vitro antitumor evaluation. (**A**) CLSM images of 4T1 cells incubated with RC NPs and Ce6 for 4 h. Scale bar: 20 μm. (**B**) FCM analysis of the intracellular uptake of RC NPs and Ce6 in 4T1 cells at different time points. (**C**) FIM images of intracellular ROS production and CLSM images obtained from 4T1 cells stained with JC-1 after different treatments. Scale bars: 10 μm and 50 μm, respectively. (**D**) Cell viability in different treatment groups at gradient concentrations of Ce6 and Rhe in the dark and (**E**) under laser irradiation. (**F**) FIM images of live/dead cell staining and (**G**) apoptotic analysis of 4T1 cells after treatment with Rhe, Ce6, or RC NPs in the presence or absence of laser irradiation. Scale bar: 10 µm. The error bars indicate means ± SD, *n* = 3, *** *p* < 0.001.

Efficient drug uptake by cells is essential for their therapeutic efficacy [37]. Subsequently, the uptake behavior of 4T1 cells toward RC NPs and free Ce6 was investigated by CLSM and flow cytometry. To better observe the temporal changes in intracellular uptake of RC NPs, CLSM images were compared at different time points. As shown in Figure S3, the red fluorescence in the cytoplasm of 4T1 cells rose progressively over time when incubated with RC NPs, suggesting effective uptake of the nanoparticles by the cells. The red fluorescence of cells treated via RC NPs was noticeably stronger than via free Ce6 after 4 h of incubation (Figure 2A), indicating that the self-assembled nanoparticles significantly increased the cellular internalization of hydrophobic drug Ce6 [38]. The results of flow cytometry also confirmed this finding (Figure 2B). Subsequently, fluorescence inverted microscopy (FIM) was employed to evaluate intracellular ROS production by RC NPs using DCFH-DA as a fluorescent probe. In Figure 2C, upon laser irradiation, 4T1 cells treated with RC NPs exhibited an obvious rise in ROS, whereas cells treated via free Ce6 exhibited very mild fluorescence [39]. This better photodynamic performance of RC NPs was mainly attributed to the enhanced drug uptake by cells as a self-assembly nanoparticle.

The excellent anti-tumor ability of RC NPs was also verified by changes in mitochondrial membrane potential (Figure 2C). In the absence of laser irradiation, RC NPs caused more reduction of mitochondrial membrane potential than free Rhe, indicated by a slightly higher green fluorescence. Upon exposure to laser irradiation, RC NPs (RC NPs+L) dramatically disrupted mitochondrial function, leading to bright green fluorescence in 4T1 cells, which might contribute to cell apoptosis [40,41]. These results confirm that the mitochondrial dysfunction mediated by RC NPs led to a large number of 4T1 cell apoptosis.

Inspired by the aforementioned results, we proceeded to examine the in vitro cytotoxicity of RC NPs using the MTT assay. In Figure 2E, the dark toxicity of the Rhe, Ce6, Rhe/Ce6, and RC NPs groups was concentration-dependent but negligible in the experimental concentrations. Once exposed to laser radiation (Figure 2F), although the concentration of Ce6 was only 0.5 μM, the RC NPs group showed a significant combination effect of PDT and chemotherapy with a cell survival rate of merely 17.8%, while the other two groups (Ce6+L and Rhe/Ce6+L) were 84.7% and 75.7%, respectively [39].

Live/dead cell staining experiments provided intuitive insights, revealing notable distinctions across groups. Figure 2G depicts the outcomes at the experimental concentration (equivalent Ce6 0.5 μM), where the majority of the cells in the RC NPs+L group exhibit bright red fluorescence (representing dead cells). Subsequently, cell apoptosis analysis (Figure 2G) showed that laser irradiation alone (PBS+L) has no effect on cell activity, and photosensitizers (Ce6+L) induced little early apoptosis (1.22%) and late apoptosis (7.01%) [9]. Of particular note is that under the same concentration of Ce6, the RC NPs + L group showed a higher rate of cell apoptosis, including 8.73% of early apoptosis and 41.4% of late apoptosis.

Based on the above results, RC NPs demonstrated excellent anti-tumor properties in vitro due to its improved cellular uptake ability and the combination therapy of PDT and chemotherapy.

### 3.3. Evaluation of US-Enhanced Tumor Penetration of RC NPs

**Figure 3:**
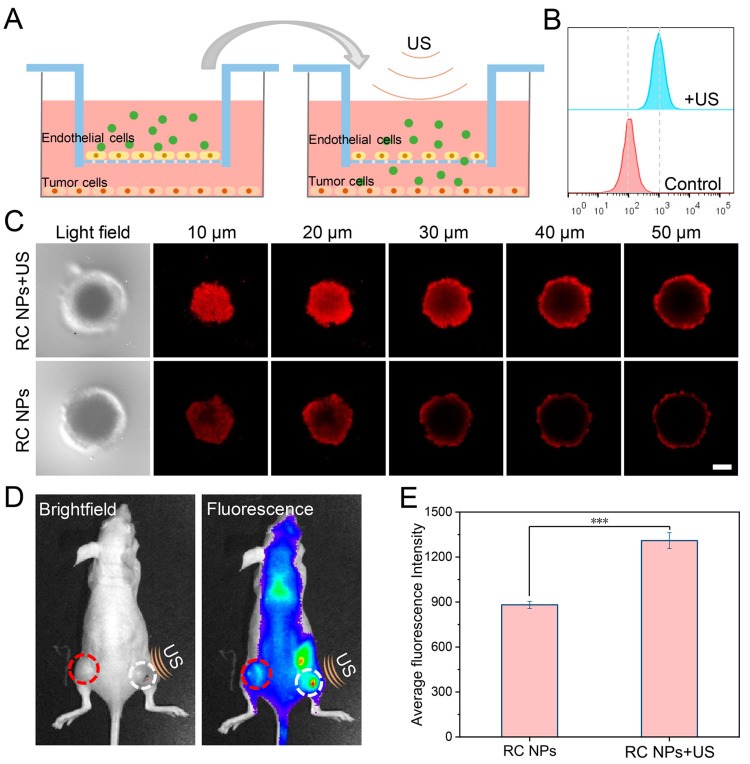

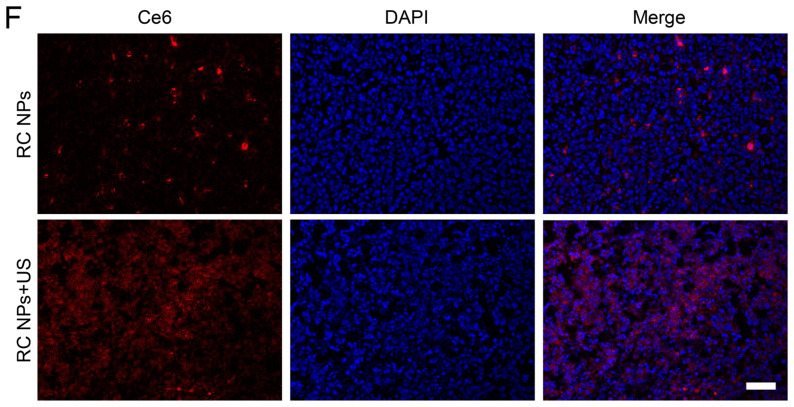
US-enhanced penetration of RC NPs. (**A**) Schematic illustration of US-promoted penetration of RC NPs across the vascular barrier. (**B**) Flow cytometric analysis of NP uptake by tumor cells across the vascular barrier after US irradiation. (**C**) Z-stack images of RC NPs penetrating inside 3D tumor spheres with or without US irradiation. Scale bar: 100 μm. (**D**) Brightfield (**left**) and fluorescence (**right**) images of mice bearing bilateral 4T1 tumors at 6 h after intravenous administration (white circle on the right indicates US irradiation treatment; red circle on the left serves as the control), and (**E**) the corresponding quantitative fluorescence analysis. (**F**) CLSM images of frozen sections of 4T1 tumors treated with US irradiation or not. Scale bar: 50 μm. The error bars indicate means ± SD, *n* = 3, *** *p* < 0.001.

The heterogenous tumor vascular network and elevated interstitial pressure [42,43] impeded the entry of nanoparticles into tumors, hence diminishing their therapeutic efficacy. Previous studies have shown that US has the ability to temporarily open the tight junctions of tumor vascular endothelial cells and increase vascular permeability, thus facilitating the penetration of nanoparticles from tumor blood vessels [31]. Additionally, the ultrasonic radiation force can push nanoparticles to penetrate deeper into the tumor’s extracellular matrix [44]. To simulate the vascular barrier in vitro, HUVEC cells were cultured in a transwell insert, as depicted in Figure 3A. Following US irradiation, RC NPs in the upper chamber could penetrate through the gap between the endothelial cells into the lower chamber. After four hours, the lower chamber’s 4T1 cells were examined for nanoparticle uptake. According to flow cytometry analysis, the drug uptake by cells in the group that received US irradiation was eight times higher compared to the control group [45] (Figure 3B). This suggests that the US can increase the permeability of tumor blood vessels, making them easier for RC NPs to penetrate. Subsequently, 4T1 cell tumor spheres were prepared, and the effect of US irradiation on the penetration of nanoparticles into tumor spheres was evaluated using Z-stack imaging of CLSM. As shown in Figure 3C, RC NPs without US irradiation were mainly accumulated on the surface of 3D tumor spheres, while RC NPs penetrated deeply into tumor tissues under US irradiation, since more Ce6 red fluorescence could be seen inside the tumor spheres in the RC NPs+U group.

Due to the complexity of tumor vessels and interstitium, the above results can only roughly simulate the effect of US on promoting the tumor penetration of RC NPs in vitro. Thus, we further validate the experimental effect of US in mice. BALB/c nude mice with bilateral 4T1 tumors were given an intravenous injection of RC NPs solution. Thirty minutes later, only the tumor on the right side was irradiated by US for ten minutes. Six hours after administration, the drug distribution in both tumors was compared by fluorescence imaging (Figure 3D). In Figure 3E, the fluorescence quantitative analysis of bilateral tumors showed that the fluorescence of the right-side tumor was about 1.5 times of the left side one [32], indicating that more RC NPs were enriched in the right tumor, which was also verified by CLSM images of frozen sections of bilateral tumors [31] (Figure 3F). The results indicate that RC NPs can be more effectively penetrated and accumulated in tumor tissues when exposed to US radiation.

### 3.4. In Vivo Antitumor Study

**Figure 4:**
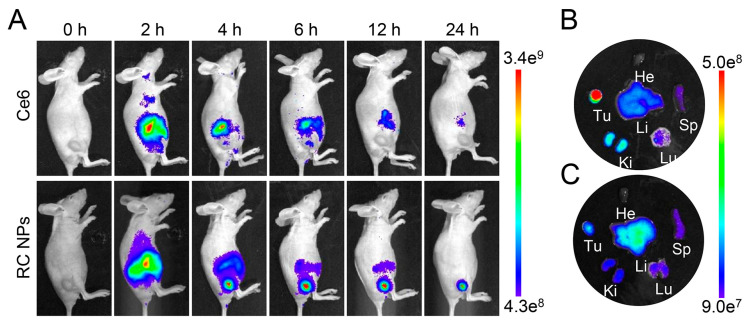

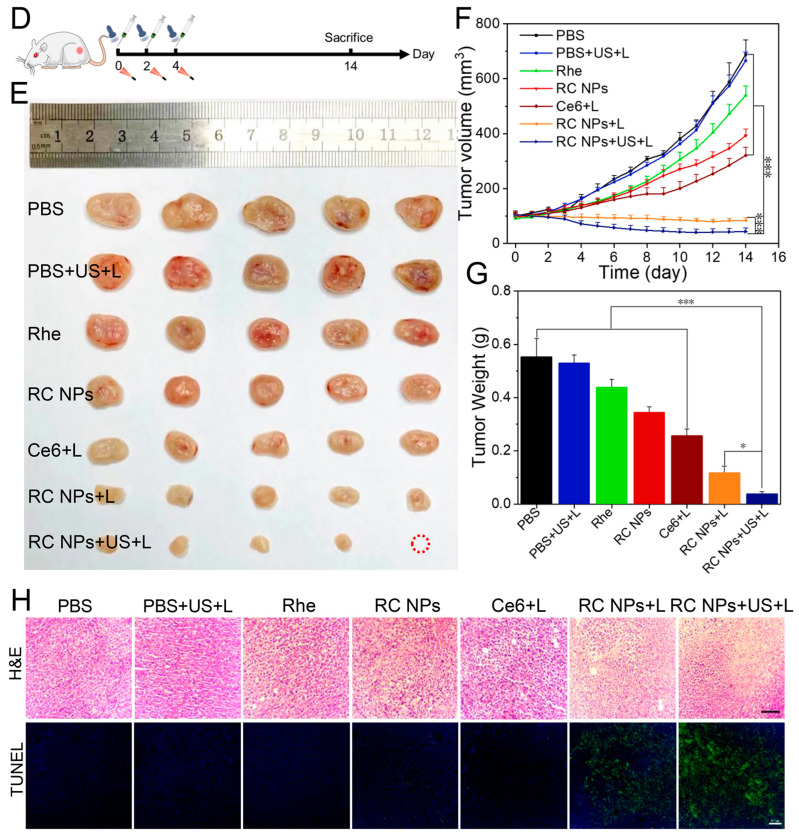
In vivo fluorescence imaging and antitumor therapy of RC NPs. (**A**) Fluorescence images in vivo at different time points after intravenous administration of RC NPs and Ce6. Fluorescence images of isolated tumor tissues and major organs 24 h after the injection of RC NPs (**B**) and Ce6 (**C**), respectively. (**D**) Schematic illustration of the treatment process. (**E**) Resected 4T1 tumor tissue(red circle represents tumor cure), (**F**) tumor growth curves, and (**G**) average tumor weight of different treatment groups. (**H**) H&E and TUNEL staining images of tumor tissues after various treatments. Scale bar: 100 μm. The error bars indicate means ± SD, *n* = 5, * *p* < 0.05, and *** *p* < 0.001.

Upon intravenous injection of the nanodrug, the photosensitizer is passively targeted to the tumor cells, enabling photodynamic therapy at the tumor location via laser irradiation [46]. The generated reactive oxygen species (ROS) are highly oxidative, capable of destroying cellular components like proteins, nucleic acids, and lipids, and producing apoptosis or necrosis [47,48]. Prior to antitumor therapy, we conducted an investigation into the tumor accumulation of RC NPs after intravenous administration by in vivo fluorescence imaging. The results illustrated in Figure 4A indicated that RC NPs exhibited a higher tendency to accumulate at the tumor site compared to free Ce6 due to the EPR effect of solid tumors [14].

Over time, the fluorescence (represented the enriched drug) in the tumor of RC NPs group reached a maximum at 6 h, then gradually decreased due to drug metabolism. Ex vivo tumor imaging (Figure 4B,C) also demonstrated that, even after a 24-h gap following drug administration, the RC NPs group’s tumor fluorescence remained 1.9 times higher than the free drug group’s (Ce6) (Figure S4) [8]. Based on the above experimental results of US-enhanced penetration of RC NPs in Figure 3, in the following tumor treatment process, we performed US and laser irradiation on mice at 0.5 h and 6 h, respectively, after intravenous treatment. The treatment process is shown in Figure 4D. Five mice per group were randomly assigned to receive PBS, PBS+U+L, Rhe, RC NPs, Ce6+L, RC NPs+L, or RC NPs+U+L after the tumor volume reached around 90 mm^3^. The tumors in the PBS+U+L group demonstrated a similar growth trend and tumor volume to the PBS group (Figure 4F), suggesting that the US and laser irradiation utilized in this experiment had no effect on tumor growth. The other groups inhibited tumor growth to a certain extent. On the 14th day, the mean tumor volumes for mice given various treatments were PBS (688.608 mm^3^), PBS+US+L (665.912 mm^3^), Rhe (539.631 mm^3^), RC NPs (393.304 mm^3^), Ce6+L (321.059 mm^3^), RC NPs+US (84.524 mm^3^), and RC NPs+US+L (43.406 mm^3^). The RC NPs group exhibited a higher tumor inhibition rate compared to the Rhe group (42.9% and 21.6%, respectively), while the RC NPs+L group demonstrated a significantly higher rate (87.7%) than the Ce6+L group (53.4%). These results indicated that EPR promoted the accumulation of RC NPs in tumors and improved the PDT therapeutic effect of Rhe and Ce6 [32]. It is especially worth emphasizing that the tumor inhibition effect in the RC NPs+U+L group was the most significant, indicating that enhanced tissue penetration and tumor accumulation by US irradiation contribute to a better therapeutic effect for RC NPs. After that, the mice tumors were taken for photography (Figure 4E) and weight. The average tumor weights of mice treated with PBS, PBS+U+L, Rhe, RC NPs, Ce6+L, RC NPs+U, and RC NPs+U+L were 0.552, 0.528, 0.438, 0.344, 0.256, 0.116, and 0.038 g, respectively (Figure 4G). The notable disparity highlights the excellent anti-tumor efficacy of the RC NPs+U+L group. In addition, H&E and TUNEL immunofluorescence staining were conducted on tumor tissues (Figure 4H). Compared with the other groups, tumor cells in the RC NPs+U+L group showed extensive nuclear shrinkage or loss of lysis and massive cell apoptosis. Taken together, RC NPs excellently exterminate tumors in vivo due to the US-mediated enhanced penetration and high drug accumulation in tumor tissues and combinational photodynamic and chemotherapy.

### 3.5. Biosafety Evaluation of RC NPs

**Figure 5:**
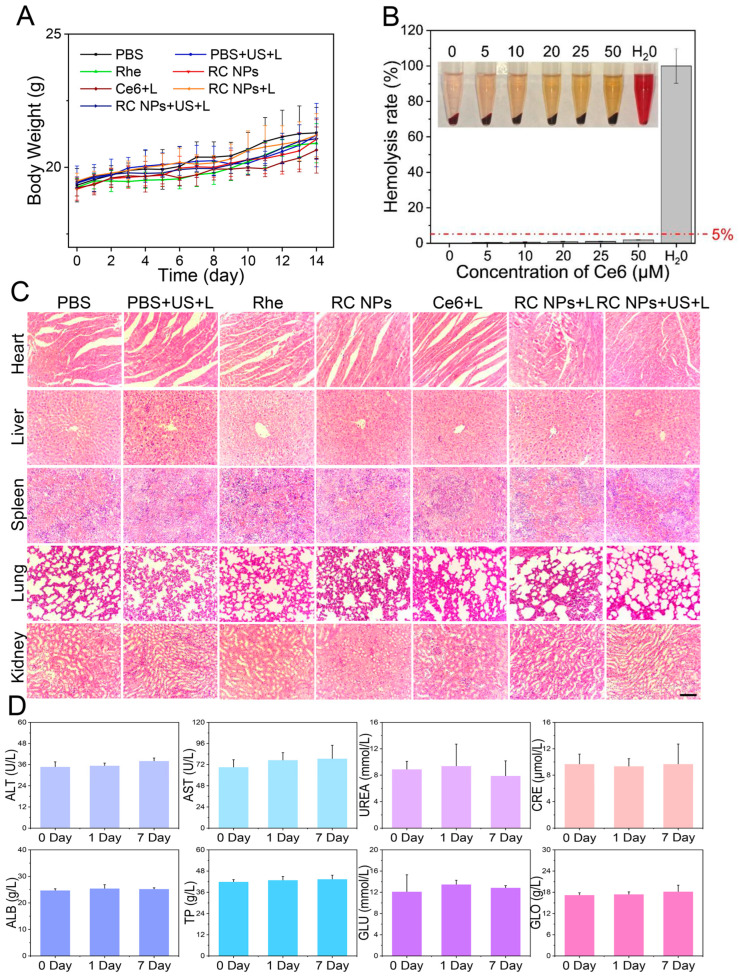
Biosafety analysis of RC NPs. (**A**) Body weight changes of mice in different treatment groups over 14 days, *n* = 5. (**B**) Changes in the hemolysis rate under different RC NP concentrations. (**C**) H&E staining analysis of major organs in different treatment groups. Scale bar: 100 μm. (**D**) Blood biochemical analysis for the biosafety evaluation of RC NPs. The error bars indicate means ± SD, *n* = 3.

We evaluated the biosafety of RC NPs by monitoring the daily body weight of mice in each group during the tumor treatment. Figure 5A illustrates the consistent stability of body weight, indicating low systemic toxicity of RC NPs. Subsequently, the biocompatibility of RC NPs was examined through the hemolysis assay. Notably, Figure 5B demonstrates that, even at the maximum drug concentration, the hemolysis rate of red blood cells remained under 5%, hence affirming its favorable blood compatibility. Subsequently, the major organs underwent H&E staining upon the completion of the treatment. In Figure 5C, no substantial organic harm was detected in the major organs, signifying that no additional damage would be caused to normal organs after intravenous administration regardless of photodynamic therapy or US, thus further confirming the safety of the in vivo application of RC NPs. Additionally, blood samples on days 1 and 7 after intravenous injection of RC NPs were taken for blood biochemical and routine tests and compared with blood samples from healthy mice, as shown in Figure 5D and Figure S5; the normal range of hematological indexes in all groups suggests that RC NPs applied in vivo are very safe for biological systems [8]. The above results indicate that the self-assembled carrier-free RC NPs not only have excellent anti-tumor effect but also have good biosafety in vivo.

## 4. Conclusions

In conclusion, we successfully prepared spindle-shaped carrier-free nanoparticles by the self-assembly of Rhe and Ce6, which have excellent stability and a very high drug loading rate. Compared to free Ce6, RC NPs were more likely to be taken up by tumor cells after self-assembly. The application of US irradiation at the tumor site facilitated the deep penetration of RC NPs, thereby significantly increasing the drug concentration within the tumor. Ultimately, the RC NPs exhibited remarkable anti-tumor efficacy under US and laser irradiation in 4T1 tumor-bearing mice.

The synthesis method of RC NPs involves self-assembly from two pure drugs, presenting a simple and safe alternative that eliminates the toxicity and immune risks associated with conventional carriers. This method is also easy to scale up for industrial production. Additionally, the use of US to enhance drug penetration is straightforward to implement in clinical practice, demonstrating significant clinical application potential. Overall, this strategy of US-enhanced penetration and accumulation of nanoparticles for the combined treatment of breast cancer offers a relatively effective option for clinical use.

## Data Availability

The original contributions presented in the study are included in the article; further inquiries can be directed to the corresponding authors.

## Supplementary Materials

The following supporting information can be downloaded at https://www.mdpi.com/article/10.3390/jfb16060206/s1. Table S1: Particle sizes and PDI values of the obtained RC NPs at different molar ratios of Rhe and Ce6. Figure S1: The changes in particle size distribution and polydispersity index (PDI) of RC NPs in (A) water, (B) PBS, and (C) 10% FBS within 7 days. Figure S2: Standard curves of (A) Ce6 and (B) Rhe detected by UV-vis spectrum and HPLC; Figure S3: CLSM images of the intracellular uptake of RC NPs in 4T1 cells at different time points, the extent of cellular uptake increased progressively with longer incubation times. Scale bar: 20 μm; Figure S4: Fluorescence quantitative analysis of major organs and tumor tissues; Figure S5: Blood routine analysis for RC NPs (*n* = 3).
