# Supplementary material for: Ultrasound-Enhanced Tumor Penetration of Carrier-Free Nanodrugs for High-Efficiency Chemo-Photodynamic Therapy of Breast Cancer
Source: J Funct Biomater. 2025 Jun 3;16(6):206. doi: 10.3390/jfb16060206 (PMC12194217; doi:10.3390/jfb16060206)
Supplement: Supplementary file 1 [file jfb-16-00206-s001.zip › jfb-3239853-supplementary.pdf]

Supporting Information

Ultrasound-enhanced Tumor Penetration of Carrier-free Nanodrugs for High-efficiency Chemo-photodynamic Therapy of Breast Cancer

Yun Xiang [a], *Shiyu Liang* [b] *and Ping Wang* [a,*]

[a] Department of Ultrasound, The Third Affiliated Hospital, Southern Medical University, Guangzhou 510630, China

[b] NMPA Key Laboratory for Research and Evaluation of Drug Metabolism & Guangdong Key Laboratory of New Drug Screening, School of Pharmaceutical Sciences, Southern Medical University, Guangzhou 510515, China

[*]Correspondence: nysycskwp123@smu.edu.cn

**Feeding ration （Rhein:Ce6）**	**5:1**	**3:1**	**1.5:1**
Average diameter(nm)	153.4±9.41	100.6±1.80	139.2±8.59
Polydispersity	0.341±0.019	0.188±0.016	0.368±0.033

Table S1. Particle sizes and PDI values of the obtained RC NPs at different molar ratios of Rhe and Ce6. The error bars indicate means ± SD, $n = 3$.

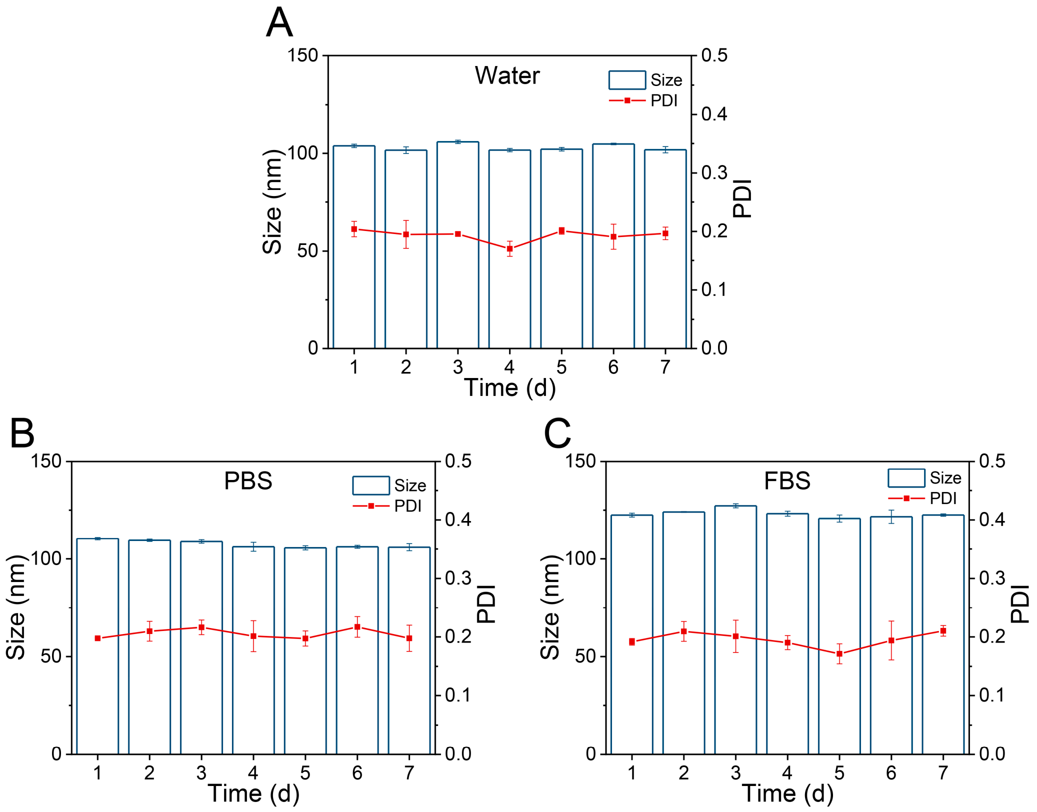

Figure S1. The changes in particle size distribution and polydispersity index (PDI) of RC NPs in (A) water, (B) PBS, and (C) 10% FBS within 7 days. The error bars indicate means ± SD, *n* = 3.

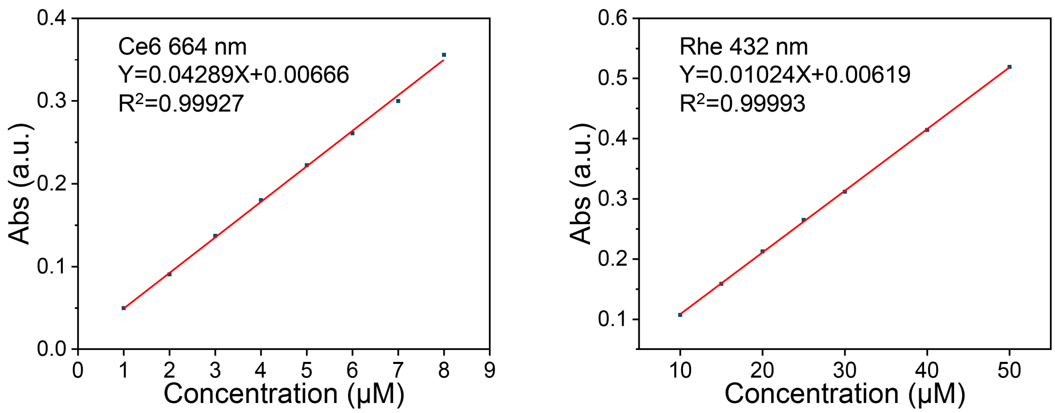

Figure S2. Standard curves of (A) Ce6 and (B) Rhe detected by UV-vis spectrum and HPLC.

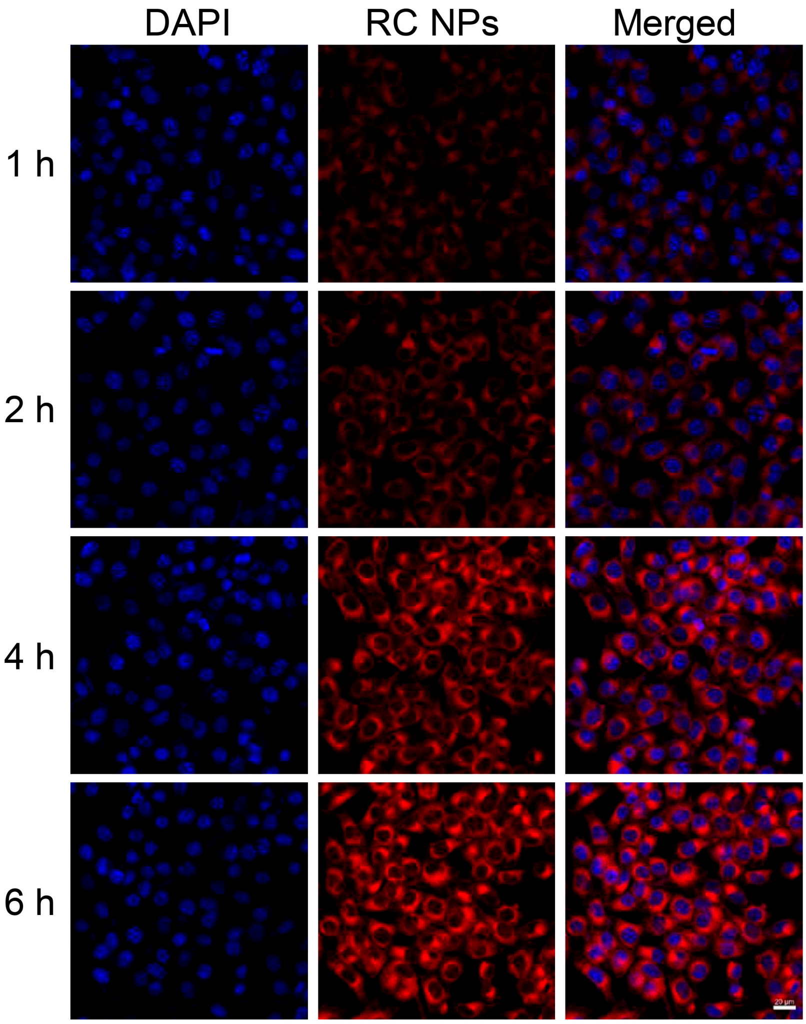

Figure S3. CLSM images of the intracellular uptake of RC NPs in 4T1 cells at different time points, the extent of cellular uptake increased progressively with longer incubation times. Scale bar: 20 μm.

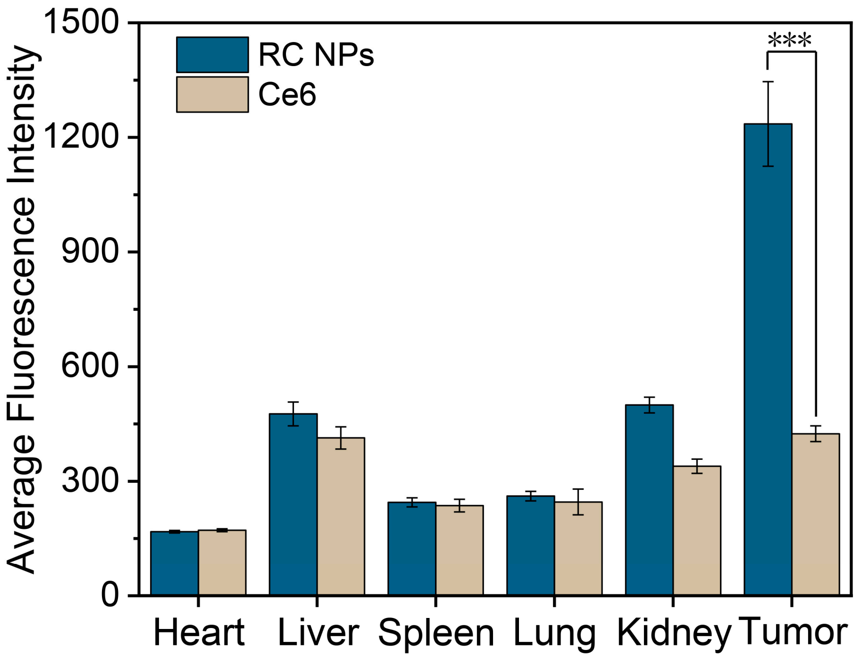

Figure S4. Fluorescence quantitative analysis of major organs and tumor tissues. The error bars indicate means ± SD, n = 3, *** p <0.001.

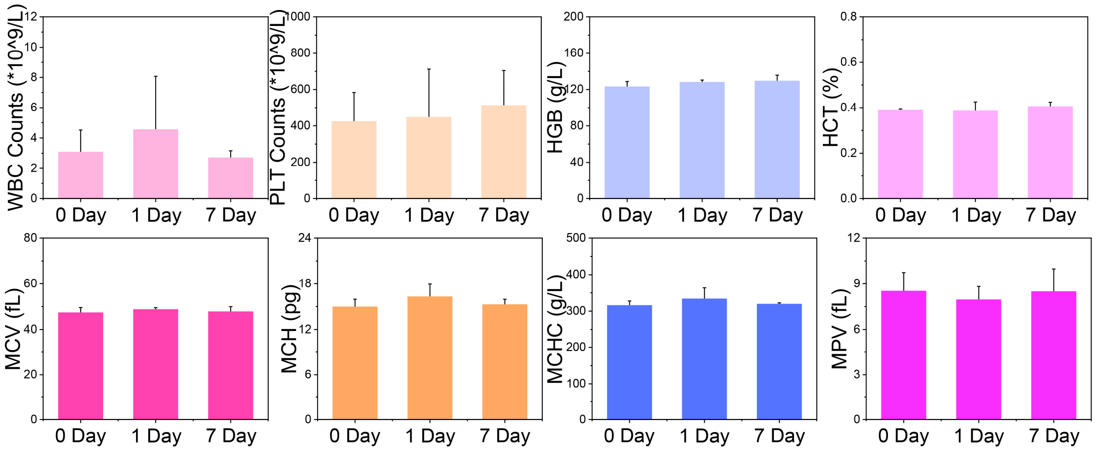

Figure S5. Blood routine analysis for RC NPs. The error bars indicate means ± SD, n = 3.
